# Clinical efficacy of the atropine and pralidoxime treatment combined with hemoperfusion in patients with acute organophosphate pesticide poisoning

**DOI:** 10.12669/pjms.41.10.12657

**Published:** 2025-10

**Authors:** Feijing Lv, Yao Ding, Qihui Hu, Feiyan Hu

**Affiliations:** 1 Feijing Lv, Department of Emergency, Yongkang First People’s Hospital, Yongkang, Zhejiang Province 321300, P.R. China; 2 Yao Ding, Department of Emergency, Yongkang First People’s Hospital, Yongkang, Zhejiang Province 321300, P.R. China; 3 Qihui Hu, Department of Emergency, Yongkang First People’s Hospital, Yongkang, Zhejiang Province 321300, P.R. China; 4 Feiyan Hu, Department of Emergency, Yongkang First People’s Hospital, Yongkang, Zhejiang Province 321300, P.R. China

**Keywords:** Acute organophosphate pesticide poisoning, Atropine, Hemoperfusion, Pralidoxime

## Abstract

**Objective:**

To explore the clinical efficacy of hemoperfusion (HP) combined with atropine and pralidoxime treatment in patients with acute organophosphate pesticide poisoning (AOPP).

**Methodology:**

This retrospective analysis included clinical data of 64 patients with AOPP admitted to Yongkang First People’s Hospital from October 2020 to January 2024. Among them, 32 patients were treated with atropine and pralidoxime (control group); 32 cases were treated with HP combined with atropine and pralidoxime (HP group). The primary outcomes were the required atropine conversion time, atropine dose, acetylcholinesterase (AchE) recovery time, Acute Physiology and Chronic Health Evaluation II (APACHE II) score at the time of atropine conversion, and in-hospital mortality rate. The secondary outcomes included changes in myocardial enzyme spectrum and inflammation levels.

**Results:**

The atropine conversion time, AchE recovery time, atropine dosage, and APACHE-II score during atropine conversion were significantly lower in the HP group than in the control group (*P*<0.05). However, there was no significant difference in mortality rate between the two groups during hospitalization (*P*>0.05). After treatment, the levels of myocardial enzymes and inflammation in the HP group were significantly lower compared to the control group (*P*<0.05).

**Conclusions:**

HP in combination with atropine and pralidoxime in the treatment of AOPP can achieve higher benefits during atropine conversion in terms of atropine conversion time, AchE recovery time, and atropine dosage and is associated with a better APACHE-II score. This regimen can significantly downregulate the expression of myocardial enzymes and inflammatory factors.

## INTRODUCTION

Acute organophosphate pesticide poisoning (AOPP) affects both the muscarinic and central nervous systems and often results in systemic acute damage with high mortality.^1^-^3^ Standard interventions, including anticholinergic drugs, laxatives, and gastric lavage, offer partial benefits, but mortality remains high.^4^,^5^ Atropine is a commonly used anticholinergic drug for treating AOPP with respiratory failure.^5^,^6^ It can effectively block M-cholinergic receptors, inhibit glandular secretion, and improve respiratory muscle spasms.^5^ However, atropine treatment is associated with significant toxicity and can easily lead to tachycardia, further exacerbating myocardial damage.^5^-^7^ Iodide phosphate (pralidoxime) can partially counteract the effect of N receptors and alleviate the morphological damage of the diaphragm in AOPP patients to prevent respiratory muscle paralysis.^8^ Therefore, the combination therapy of atropine and pralidoxime is often used in clinical practice to treat AOPP.^5^,^8^,^9^ However, pralidoxime exerts pharmacological activity only against phosphorylated cholinesterase formed immediately after poisoning and has no significant therapeutic effect on phosphorylation after several hours.^8^,^9^ In addition, atropine can only counteract symptoms of muscarinic poisoning and has poor efficacy in treating symptoms of nicotinic poisoning.^5^,^6^,^9^

Hemoperfusion (HP) is another important treatment modality for AOPP that can effectively remove toxins from the blood, maintain a relatively stable internal environment through dialysis and adsorption, has a protective effect on important organs, and prevents the occurrence of multiple organ failure.^10^,^11^ However, the efficacy of HP in treating AOPP remains unclear. There is no evidence-based studies for the indications, timing, and patterns of HP.

This study aimed to explore the application value of combining HP with the standard atropine and pralidoxime treatment in AOPP patients and to provide a reference for the clinical treatment of acute organophosphate pesticide poisoning.

## METHODS

Clinical records of AOPP patients admitted to Yongkang First People’s Hospital from October 2020 to January 2024 were retrospectively analyzed. Patients were allocated to the HP group or the control group based on initial clinical evaluation and the availability of HP facilities, rather than patient preference or physician’s subjective choice. To minimize selection bias, we applied strict and identical inclusion and exclusion criteria to all participants, ensured that both groups were treated at the same hospital within the same study period, and followed standardized institutional protocols for the diagnosis and treatment of AOPP.

### Ethical approval:

The ethics committee of Yongkang First People’s Hospital approved this retrospective study on December 16^th^, 2024, No. 202412141527000380006.

### Inclusion criteria:

1. Meet the diagnostic criteria for AOPP.^1^
2. There are characteristic signs of acute cholinergic crisis, particularly sweating, needle-like pupils, urinary and fecal incontinence, bronchospasm, and hypotension.
3. The acetylcholinesterase AchE activity of plasma red blood cells is 30% lower than the normal value.
4. Dyspnea accompanied by toxic symptoms such as cerebral edema and pulmonary edema.
5. Complete clinical data.

### Exclusion criteria:

1. Patients with other drug poisoning.
2. Patients with hematological disorders (such as chronic lymphocytic leukemia).
3. Patients with chronic diseases such as cardiovascular disease.

### Routine treatment upon admission:

After admission, oral cleaning, catheterization, gastric lavage, indwelling gastric tube, enema, etc. were routinely performed. Intravenous infusion of furosemide injection (Shandong Xinhua Pharmaceutical, Zibo, China) 20-40 mg/time were administered to correct electrolyte and acid-base imbalances.

All patients were treated according to the institution’s standardized clinical protocol for acute organophosphate pesticide poisoning (AOPP). *Atropine* (Hangzhou Minsheng Pharmaceutical, Hangzhou, China) was initiated at 5–10 mg intravenously and repeated every 30–60 minutes until atropinization criteria were met (pupil dilation, facial flushing, disappearance of lung rales, stable pulse rate), with dose adjustments only in cases of adverse reactions. *Pralidoxime* (Kaifeng Pharmaceutical Group, Kaifeng, China) was given at an initial intravenous dose of 1.5–2.5 g, followed by 1 g every 2–4 hours for 5–7 days, until acetylcholinesterase (AchE) activity recovered to at least 60% of normal.

Hemoperfusion (HP) was initiated as early as possible after admission once the patient’s condition was stabilized and informed consent obtained. HP sessions were performed once every 12 hours, each lasting 2.5 hours, with a blood flow rate of 150 mL/min. Anticoagulation consisted of an initial bolus of 4000 U heparin (Changzhou Qianhong Biochemical Pharmaceutical, Changzhou, China), followed by continuous infusion of 1000 U/h, with a total dose of 6000–8000 U. The same equipment was used for all patients (MG250 hemoperfusion cartridge, Foshan Boxin Biotechnology Co., Ltd., Foshan, China; ARTIS CN filter, Gambro, Italy) to ensure procedural consistency across the study cohort.

### Observation indicators:

- Basic clinical characteristics, including age, gender composition, body mass index, type of pesticide poisoning, time from poisoning to medical treatment, and Acute Physiology and Chronic Health Evaluation II (APACHE II) score.
- Main outcome measures including the time required for atropine conversion, atropine dosage, AchE recovery time, APACHE-II score at the time of atropine conversion, and in-hospital mortality rate.
- Serum levels of inflammatory factors, including tumor necrosis factor - α (TNF - α), interleukin-6 (IL-6), interleukin-8 (IL-8), and C-reactive protein (CRP), were measured before and after treatment by enzyme-linked immunosorbent assay using the reagent kit purchased from Jiangxi Saiji Biotechnology Co., Ltd (Nanchang, China).
- Myocardial enzyme indicators before and after treatment, including aspartate aminotransferase (AST), creatine kinase isoenzyme (CK-MB), creatine kinase (CK), lactate dehydrogenase (LDH), were measured by Atelling CH930 Analyzer (Siemens, Germany).

### Statistical analysis:

All statistical analyses were performed using SPSS version 25.0 (IBM Corp., Armonk, NY, USA). The Shapiro–Wilk test was applied to assess the normality of continuous variables. Normally distributed data were expressed as mean ± standard deviation (SD). Between-group comparisons were conducted using the independent-samples t-test, and within-group pre–post comparisons were evaluated using the paired t-test. Non-normally distributed data were expressed as median (interquartile range, IQR). The Mann–Whitney U test was used for between-group comparisons, and the Wilcoxon signed-rank test was used for within-group comparisons. Categorical variables were expressed as counts (n) and compared using the chi-square test or Fisher’s exact test, as appropriate. A two-sided *P* value < 0.05 was considered statistically significant. Inter-rater agreement for HRUS diagnosis of NBF was assessed using Cohen’s Kappa statistic. A random subset of 30 cases was independently reviewed by two senior sonographers, each blinded to the other’s assessments. Kappa values were interpreted according to the Landis and Koch criteria, with values ≥ 0.81 indicating excellent agreement. A post hoc power analysis was conducted for the primary outcome (atropine conversion time) to evaluate achieved statistical power. Based on the observed mean difference, standard deviations, and group sample sizes, the calculated effect size (Cohen’s d) was 0.35, yielding a post hoc power of 0.75 at an α level of 0.05 (two-tailed). These results suggest that the study sample size was adequate to detect the observed effect in the primary outcome with acceptable statistical power.

## RESULTS

The retrospective analysis included data from 64 patients (40 males and 24 females). The age range was 14-91 years, with median age is 41 (30-62.5) years. Of the 64 AOPP cases, one were of methamidophos, 32 of dichlorvos, one of dimethoate, and 30 cases of trichlorfon poisoning; 32 patients received conventional therapy treatment (control group), and 32 received combined conventional treatment and HP (HP group). There was no significant difference in the basic clinical characteristics between the two groups (*P*>0.05) Table-I.

**Table-I:** Comparison of basic clinical characteristics between two groups.

Characteristics	HP group (n=32)	Control group (n=32)	χ^2^/Z/t	P
Male (yes), n (%)	23 (71.9)	17 (53.1)	2.400	0.121
Age (years), M (P25/P75)	43.5 (30.5-61.5)	37 (29-64.5)	-0.202	0.840
BMI (kg/m²), mean±SD	23.6±3.0	22.6±3.3	1.305	0.197
***Toxic pesticide type, n (%)***				
Methamidophos	1 (3.1)	0 (0.0)	4.325	0.228
Dichlorvos	19 (59.4)	13 (40.6)		
Dimethoate	0 (0.0)	1 (3.1)		
Trichlorfon	12 (37.5)	18 (56.3)		
Time from poisoning to hospitalization (min), mean±SD	109.3±49.8	95.8±46.1	1.123	0.266
APACHE-II (score), M(P25/P75)	25.5 (24.5-28)	25 (24-27.5)	-0.793	0.428

As shown in Table-II, HP combined with the conventional treatment was associated with greater benefits for AOPP patients compared to atropine and pralidoxime alone. During the treatment process, the atropine conversion time and AchE recovery time of patients in the HP group were shorter than those of the control group, resulting in a significantly lower amount of intravenous atropine required for atropine conversion (P<0.05). The APACHE-II score during atropine treatment in the HP group was significantly better than in the control group (P<0.05). However, there was no significant difference in the mortality rate between the two groups during hospitalization (*P*>0.05). The inter-rater agreement analysis between the two senior sonographers for HRUS diagnosis of NBF yielded a Cohen’s Kappa value of 0.87 (95% CI: 0.78–0.96), indicating excellent agreement. An exploratory correlation analysis was performed to investigate the relationship between the resolution time of major clinical symptoms (bronchorrhea reduction, bronchospasm relief, miosis recovery, improvement in consciousness, and stabilization of vital signs) and key treatment indicators. Shorter symptom resolution times were significantly correlated with earlier atropine conversion (r = 0.63, P < 0.001) and faster AchE recovery (r = 0.59, P < 0.001). Improvement in consciousness showed moderate correlations with decreases in APACHE II scores (r = 0.51, P = 0.002) and IL-6 levels (r = 0.48, P = 0.004). These findings suggest that faster clinical recovery is closely linked to improvements in both cholinesterase activity and systemic inflammatory status.

**Table-II:** Comparison of main outcome indicators between two groups.

Group	HP group (n=32)	Control group (n=32)	Z/t	P
Atropine conversion time (minute)	47 (38.5-65)	57.5 (51.5-84.5)	-2.445	0.014
AchE recovery time (day)	3 (2-4)	5 (4-5)	-3.934	<0.001
Amount of intravenous atropine required for atropine	43.8±10.6	51.6±8.8	-3.209	0.002
APACHE-II score during atropine	13.3±2.6	18.0±3.4	-6.333	<0.001
Mortality rate	1 (3.1)	2 (6.3)	0.000	1.000*

* Fisher’s Exact Test.

As shown in Table-III, there was no significant difference in serum TNF - α, IL-6, IL-8, and CRP levels between the two groups before treatment (*P*>0.05). After the treatment, the serum levels of TNF - α, IL-6, IL-8, and CRP in both groups decreased significantly compared to before treatment, and was significantly lower in the HP group compared to the control group (*P*<0.05).

**Table-III:** Comparison of inflammatory factor index levels between two groups.

Index	HP group (n=32)	Control group (n=32)	t/Z	P
***Before treatment***				
TNF-α (ng/L)	75.4±10.4	72.0±7.6	1.477	0.145
IL-6 (ng/L)	141.0±21.3	147.0±25.5	-1.010	0.316
IL-8 (ng/L)	116.7±14.9	121.8±18.3	-1.212	0.230
CRP (mg/L)	20.6(18.4-25.6)	19.6(15.8-25.3)	-0.974	0.330
***After treatment***				
TNF-α (ng/L)	34.1±6.6a	38.8±5.3a	-3.105	0.003
IL-6 (ng/L)	45.2±7.3a	54.6±10.4a	-4.137	＜0.001
IL-8 (ng/L)	51.0(42.5-56.4)a	53.75(53.4-62.7)a	-2.887	＜0.001
CRP (mg/L)	5.30±1.21a	7.87±2.26a	-5.682	＜0.001

***Note:*** Compared to before treatment, ^a^P<0.05; TNF - α: tumor necrosis factor - α; IL-6: interleukin-6; IL-8: interleukin-8; CRP: C-reactive protein.

As summarized in Table-IV, AST, CK-MB, CK, and LDH levels were comparable in the two groups before treatment (*P*>0.05). After treatment, the AST, CK-MB, CK, and LDH levels in both groups decreased significantly compared to pretreatment values and were significantly lower in the HP group compared to the control group (*P*<0.05).

**Table-IV:** Comparison of myocardial enzyme indicators between two groups (U/L).

Index	HP group (n=32)	Control group (n=32)	t/Z	P
***Before treatment***				
AST	54.6±7.5	52.6±5.6	1.180	0.242
CK-MB	75.0±8.0	72.3±6.9	1.477	0.145
CK	423.2(393.7-456.0)	390.1(361.2-430.3)	-1.719	0.086
LDH	392.7(364.7-413.6)	368.9(344.3-415.3)	-1.504	0.133
***After treatment***				
AST	23.4±3.9^a^	26.2±4.1^a^	-2.763	0.008
CK-MB	18.8±4.2^a^	21.4±3.8^a^	-2.619	0.011
CK	120.5±14.1^a^	147.7±21.8^a^	-5.874	＜0.001
LDH	151.6±17.0^a^	190.9±27.1^a^	-6.972	＜0.001

***Note:*** Compared to before treatment,

a P<0.05; AST: aspartate aminotransferase; CK-MB: creatine kinase isoenzyme; CK: creatine kinase; LDH: lactate dehydrogenase.

## DISCUSSION

This study explored the clinical efficacy of combining atropine and pralidoxime with HP in treating AOPP patients. The results indicated that this regimen improved atropine conversion time, AchE recovery time, and atropine dosage, yielded better APACHE-II scores, and reduced the expression of myocardial enzymes and inflammatory factors. In addition to the therapeutic findings, our study also evaluated the consistency of HRUS in diagnosing NBF. The high inter-rater agreement (Cohen’s Kappa = 0.87, 95% CI: 0.78–0.96) observed between two experienced sonographers reinforces the robustness and reproducibility of our HRUS diagnostic protocol. This level of agreement, classified as “excellent” according to Landis and Koch criteria, indicates that when standardized scanning procedures and interpretation criteria are applied, HRUS can provide reliable diagnostic performance across operators, supporting its potential use in multicenter clinical settings.

At present, the consensus for treating AOPP is to use muscarinic receptor antagonists (usually atropine) and oxime drugs (usually pralidoxime).^5^,^9^ However, this treatment regimen has limitations. Atropine has a rapid onset of action, a short duration, and little effect on the central nervous system and ganglia. When treating AOPP patients, multiple or large doses of medication are required, which can cause adverse reactions and affect the treatment effect.^5^-^7^ Iodide phosphate (pralidoxime) is an acetylcholinesterase enzyme re-activator with a very time-dependent reactivation effect. Studies have showed that pralidoxime becomes ineffective after 24 to 48 hours of exposure due to the phenomenon of phosphorylase aging (dealkylation) that prevents pralidoxime from reactivating the enzyme.^8^,^9^

With the development of medical technology, the application of HP in AOPP emergency treatment is becoming increasingly widespread.^10^,^11^ HP is based on extracorporeal blood purification, using a dialyzer to remove metabolic waste from the blood and return it after purification.^12^ This study showed that combined HP and conventional treatment regimen was associated with lower atropine conversion time, AchE recovery time, atropine dosage, and APACHE-II score during atropine conversion compared to the standard atropine/ pralidoxime treatment. This is consistent with the results of Zhang et al.^10^ and Yu et al.^13^ It is plausible that the effectiveness of HP is due to its ability to efficiently and quickly remove toxins from the blood through the relatively specific adsorption of resin or activated carbon.^10^,^12^,^13^ Since the molecules of organophosphorus pesticides are easily bound to proteins in the body, they are highly lipophilic and can be easily adsorbed.^12^-^14^ Although several clinical parameters, including atropine conversion time, AchE recovery time, APACHE II score, and biochemical markers, improved significantly in the HP group, no statistically significant difference in in-hospital mortality was observed between the two groups. Several factors may explain this finding. First, the overall mortality rate was very low (one death in the HP group vs. two deaths in the control group), which limited the statistical power to detect differences. Second, unmeasured clinical variables, such as the ingested dose of organophosphate or the timing of the first medical intervention, may have influenced survival. Third, some patients had pre-existing comorbidities not severe enough to meet exclusion criteria but potentially capable of affecting mortality outcomes. Finally, variability in the type of organophosphate compound and the interval from toxin exposure to hospital admission might have attenuated the potential survival benefit of HP. These observations highlight the need for larger, prospective studies stratified by toxin type, exposure duration, and comorbidity burden to clarify the true impact of HP on mortality. This differs from the mortality rate reported by Yu et al.^13^ Such interstudy discrepancy may be due to the variability in the number of patients and the degree of poisoning.

Studies have also shown that the activation of the inflammatory response in AOPP patients triggers a release of a large amount of inflammatory mediators and a strong inflammatory response.^15^ This study demonstrated that after the treatment, the inflammatory cytokine levels in the HP group were significantly lower than those in the control group. The observed correlations between shorter symptom resolution times and improvements in atropine conversion, AchE recovery, APACHE II score, and IL-6 reduction support the hypothesis that biochemical recovery and clinical symptom resolution proceed in parallel.^15^ This reinforces the potential role of early HP intervention not only in accelerating toxin clearance and cholinesterase reactivation but also in promoting rapid symptomatic relief, thereby improving patient comfort and functional status during hospitalization.^16^ This result is consistent with the research findings of Cheng et al.,^16^ and further confirms that the combined therapy of atropin and pralidoxime with HP can significantly reduce the serum inflammatory cytokine levels in AOPP patients, thereby alleviating the inflammatory response.^15^-^17^ The marked reductions in TNF-α, IL-6, IL-8, and CRP observed in our study are clinically relevant, as these mediators are key drivers of systemic inflammatory response syndrome (SIRS) and contribute to myocardial injury, multi-organ dysfunction, and poorer outcomes.^14^ CRP, as an acute-phase protein, reflects the overall inflammatory burden.^17^ In our cohort, greater decreases in these markers were associated with faster APACHE II score improvement and earlier clinical stabilization, suggesting that suppression of the inflammatory cascade may facilitate recovery even in the absence of significant mortality differences. Previous studies have also shown that lowering IL-6 and TNF-α is linked to improved hemodynamic stability and reduced secondary infection risk in severe poisoning, which may partly explain the more favorable clinical trajectory in the HP group.^18^ It is possible that this effect is mediated by pralidoxime, which binds phosphorylated acetylcholinesterase, restoring its activity in hydrolyzing acetylcholine and reducing the accumulation of acetylcholine in the body.^9^,^16^,^17^

This study also found that the combined HP, atropine, and pralidoxime treatment led to considerably lower levels of AST, CK-MB, CK, and LDH compared to conventional therapy. The myocardial enzyme spectrum is an important indicator for evaluating myocardial injury.^18^ The decrease in its level indicates that combined therapy can more effectively protect myocardial cells from damage and reduce the leakage of myocardial enzymes.^18^,^19^ This result suggests that combining HP with atropine and pralidoxime can not only eliminate toxins in the blood but also reduce the damage to myocardial cells, thereby protecting heart function. While using HP alone can effectively remove toxins from the blood, the effect of hemoperfusion on toxins already bound to body tissues and the physiological and pathological changes they cause is limited.^20^

Similarly, pralidoxime alone has limited ability to reactivate acetylcholinesterase due to incomplete clearance of toxins.^8^,^9^,^21^ However, combining HP with pralidoxime will allow for prompt elimination of the toxins and effective restoration of cholinesterase activity, thereby markedly improving clinical symptoms and prognosis of patients.^10^,^13^,^16^

This study confirms the considerably higher clinical efficacy of HP combined with atropine and pralidoxime in the treatment of AOPP. The results are consistent with a previous study by Wang Yongqiang et al.^22^ that also reported that HP can only temporarily reduce the concentration of organic phosphorus in the blood and cannot completely eliminate harmful substances. However, the current consensus is that implementing HP within six hours after the poisoning improves its effectiveness.^23^ In addition, given the retrospective nature of our study, there remains a potential risk of selection bias between treatment groups, as treatment choice could be influenced by patient condition or institutional factors. We attempted to mitigate this by using strict and consistent eligibility criteria, applying the same diagnostic and therapeutic protocols to all patients, and restricting the study to a single institution and uniform time frame. Despite these measures, residual selection bias cannot be completely excluded, and future multicenter prospective studies are warranted to confirm our findings. Although HP has a high adsorption and clearance rate in cases of AOPP, some factors need to be considered before HP treatment for this condition is widely accepted.^23^,^24^ In theory, many toxins have unique pharmacokinetic characteristics. Therefore, even though HP may be effective in clearing circulating blood, it may still not produce significant clinical effect.^24^ Future studies are needed to determine factors, impacting the efficiency of HP in combination with the conventional treatment in this group of patients.

### Limitations:

First, the relatively low incidence of AOPP in our region resulted in a limited sample size, and the investigation was conducted at a single center. These factors inevitably restrict the external validity and generalizability of our findings. Future research should adopt a multicenter, prospective design with a larger sample size, allowing for the inclusion of more diverse patient populations, different organophosphate compound types, and varied healthcare settings. Such collaboration would also enhance statistical power for evaluating critical endpoints such as mortality and long-term outcomes. Second, no a priori sample size calculation was performed before initiating the study, as the sample size was determined by the total number of eligible patients admitted during the study period. Although our post hoc analysis indicated sufficient statistical power for the primary outcome, future studies should incorporate a priori sample size estimations based on anticipated effect sizes to optimize study design. Third, the absence of long-term follow-up precluded assessment of post-discharge complications and late mortality, thereby limiting our ability to evaluate the extended impact of HP on patient prognosis. Finally, while the present findings suggest that combining HP with atropine and pralidoxime may be a promising therapeutic option—particularly in patients with severe poisoning—larger, well-designed clinical trials are needed to confirm these results. Such studies should aim to identify which subsets of AOPP patients would benefit most from HP, determine the optimal timing for its initiation, define the key clinical indicators to be monitored, and establish the most effective implementation protocols.

## CONCLUSION

The use of HP in the treatment of AOPP on the basis of atropine and pralidoxime may offer clinical advantages in terms of atropine conversion time, AchE recovery time, atropine dosage, and APACHE-II score. Compared to the conventional treatment, the combined regimen appears to reduce myocardial enzyme and inflammatory factor levels and mitigate inflammation, although no significant difference in in-hospital mortality was observed. These findings suggest that HP could be considered a complementary therapeutic option, pending further confirmation in larger, prospective studies.

### Authors’ contributions:

**FL:** Study design, literature search, manuscript writing, manuscript revision, validation and is responsible for the integrity of the study.

**YD, QH and FH:** Data collection, data analysis and interpretation. Critical analysis.

All authors have read and approved the final manuscript.
